# Neuromedin U Deficiency Disrupts Daily Testosterone Fluctuation and Reduces Wheel-Running Activity in Rats

**DOI:** 10.1210/endocr/bqaf102

**Published:** 2025-06-05

**Authors:** Mai Otsuka, Yu Takeuchi, Maho Moriyama, Sakura Egoshi, Yuki Goto, Tingting Gu, Atsushi P Kimura, Shogo Haraguchi, Taishi Yoshii, Sakae Takeuchi, Makoto Matsuyama, George E Bentley, Sayaka Aizawa

**Affiliations:** Graduate School of Natural Science and Technology, Okayama University, Okayama 700-8530, Japan; Graduate School of Natural Science and Technology, Okayama University, Okayama 700-8530, Japan; Graduate School of Natural Science and Technology, Okayama University, Okayama 700-8530, Japan; Department of Biology, Faculty of Science, Okayama University, Okayama 700-8530, Japan; Graduate School of Natural Science and Technology, Okayama University, Okayama 700-8530, Japan; Graduate School of Environmental, Life, Natural Science and Technology, Okayama University, Okayama 700-8530, Japan; Department of Biological Sciences, Faculty of Science, Hokkaido University, Sapporo 060-0810, Japan; Department of Biochemistry, Showa University School of Medicine, Tokyo 142-8555, Japan; Graduate School of Natural Science and Technology, Okayama University, Okayama 700-8530, Japan; Department of Biology, Faculty of Science, Okayama University, Okayama 700-8530, Japan; Graduate School of Environmental, Life, Natural Science and Technology, Okayama University, Okayama 700-8530, Japan; Graduate School of Natural Science and Technology, Okayama University, Okayama 700-8530, Japan; Department of Biology, Faculty of Science, Okayama University, Okayama 700-8530, Japan; Graduate School of Environmental, Life, Natural Science and Technology, Okayama University, Okayama 700-8530, Japan; Division of Molecular Genetics, Shigei Medical Research Institute, Okayama 701-0202, Japan; Department of Integrative Biology and Helen Wills Neuroscience Institute, University of California at Berkeley, Berkeley, CA 94720, USA; Graduate School of Natural Science and Technology, Okayama University, Okayama 700-8530, Japan; Department of Biology, Faculty of Science, Okayama University, Okayama 700-8530, Japan; Graduate School of Environmental, Life, Natural Science and Technology, Okayama University, Okayama 700-8530, Japan; Department of Integrative Biology and Helen Wills Neuroscience Institute, University of California at Berkeley, Berkeley, CA 94720, USA

**Keywords:** Neuromedin U, rat, motivation, activity, testosterone, wheel-running

## Abstract

The objective of this study was to elucidate the role of endogenous Neuromedin U (NMU) in rats by performing NMU knockout (KO). Male, but not female NMU KO rats exhibited decreased wheel-running activity vs wildtype (WT), although overall home cage activity was not affected. Plasma testosterone in WT rats varied significantly over the course of a day, with a peak at ZT1 and a nadir at ZT18, whereas in NMU KO rats testosterone remained stable throughout the day. Chronic administration of testosterone restored wheel-running activity in NMU KO rats to the same level as in WT rats, suggesting that the decrease in wheel-running activity in NMU KO rats is due to the disruption of the diurnal change of testosterone. Accordingly, expression of the luteinizing hormone beta subunit (*Lhb*) mRNA in the pars distalis of anterior pituitary was significantly lower in NMU KO rats; immunostaining revealed that the size of luteinizing hormone (LH)–expressing cells was also relatively small in those animals. In the brain of male WT rats, *Nmu* was highly expressed in the pars tuberalis, and the NMU receptor *Nmur2* was highly expressed in the ependymal cell layer of the third ventricle. This study reveals a novel function of NMU and indicates that endogenous NMU in rats plays a role in the regulation of motivated activity via regulation of testosterone.

Neuromedin U (NMU) is a peptide hormone first identified in the porcine spinal cord in 1985 (1), and which is now known to be present in various species of mammals, birds, amphibians, and fish (2-6). In mammals, NMU is highly expressed both centrally and peripherally in the brain, pituitary gland, gastrointestinal tract, and moderately expressed in the testis, ovary, thyroid gland, spleen, lymphocytes, adipose tissue, endothelial cells, and pancreas (7-10). This broad distribution suggests that NMU has multiple physiological functions, to wit feeding and energy expenditure (11), stress responses (12), circadian rhythmicity (13), tumorigenesis (14), and inflammation (15, 16). Furthermore, to investigate the physiological and behavioral roles of endogenous NMU by genetic analyses, NMU-deficient mice were generated and developed hyperphagia and obesity (17). By contrast, transgenic mice overexpressing NMU are hypophagic and leaner than wildtype (WT) mice (18). The phenotypes of genetically modified mice strongly support a role of NMU in energy homeostasis in mice. Despite these reports in mice, inconsistent results have been obtained from administration experiments, especially in rats. Intracerebroventricular injection of NMU into fasted rats decreases subsequent food intake and body mass gain, but not in free-feeding rats (19). It has also reported that chronic injection of NMU into the paraventricular nucleus (PVN), which contains the NMU receptor, of free-feeding rats does not influence food intake or body mass (20). Finally, we recently reported that NMU knockout (KO) in rats did not cause hyperphagia or hypertrophy (21). Our data suggest that, unlike in mice, the main function of endogenous NMU in rats may be distinct from that of feeding control.

The cause of discrepancies in the phenotype of NMU KO in mice vs rats may be due to species-specific differences in the expression regions of NMU and NMU receptors. In the mouse hypothalamus, *Nmu* is expressed in the arcuate nucleus (ARC), dorsomedial hypothalamic nucleus (DMH), and ventromedial hypothalamic nucleus (VMH), and NMU receptor type 2 (*Nmur2*) is expressed in the PVN, DMH, VMH, and ARC (22); those are well-known regions involved in feeding and energy balance control. However, in rats, *Nmu* and *Nmur2* mRNAs in the same hypothalamic nuclei were hardly detectable (21). Instead, in rats, *Nmu* is highly expressed in the pars tuberalis and *Nmur2* is highly expressed in the ependymal cell layer of the third ventricle) (21, 23). Graham et al (22) reported important differences in *Nmu* and *Nmur2* expression sites between the mouse and rat hypothalamus (24) by in situ hybridization (ISH) and they demonstrated that *Nmu* mRNA is abundantly expressed in the mouse DMH and VMH, but only weakly in the rat DMH and not in the VMH. Thus, based on neuroanatomical studies, it appears reasonable that NMU deficiency in rats impairs neither body mass nor food intake. Thus, although the peptide sequence of the NMU c-terminal (active site) is highly conserved among species, the species-specific expression pattern may allow NMU to have distinct functions across species.

The objective of this study was to investigate the role of endogenous NMU in rats. Based on prior research suggesting NMU involvement in the regulation of activity (22, 25, 26), our study was particularly focused on activity. For this purpose, we analyzed NMU KO rats and found that they exhibited a significant decrease in wheel-running activity but not overall home cage activity. Additionally, the diurnal change in plasma testosterone concentration seen in male WT rats was nonexistent in male NMU KO rats. These findings suggest that endogenous NMU in male rats may play a role in regulating circulating testosterone and thereby enhancing motivation for wheel-running activity.

## Materials and Methods

### Animals

NMU KO rats were previously generated from F344 rats obtained from Charles River Laboratories Japan, Inc. (Kanagawa, Japan), by the rGONAD method and CRISPR-Cas9 system as described previously (21). WT and NMU KO rats used in this study were generated by crossing *Nmu*+/– rats. The rats were maintained in a 12-hour light/dark cycle (light switched on at 08:00) at room temperature (23 ± 2 °C) with food and water provided ad libitum. Animal experiments were approved by the Animal Care and Use Committee at Okayama University (permission number: OKU-2020016, OKU-2023088, OKU-2023351, OKU-2023350) and were performed in accordance with the Guidelines for Animal Experimentation at Okayama University. All efforts were made to minimize animal suffering and to reduce the number of animals used in the experiments. Reporting of animal data in this study followed the recommendations set out in the ARRIVE guidelines.

Genotyping was performed by polymerase chain reaction with genomic DNA isolated from an ear piece. PCR amplification was performed using EmeraldAmp MAX PCR Master Mix (Takara Bio, Inc., Shiga, Japan) in accordance with the manufacturer's instructions. Oligonucleotide-specific primers for WT and KO alleles were Nmu-Fw (5′-GATTTAAAAGTTGGTGGCGCG-3′) and Nmu-Rv (5′-GACAGGAGAGGAGATGCAGTT-3′). Amplicon sizes were confirmed by 2% agarose gel electrophoresis (product sizes: WT allele, 222 bp; KO allele; 242 bp).

### Home Cage Activity

Locomotor activity was measured using the E-mitter telemetry system (Starr Life Sciences Corp., PA, USA). Rats were housed in a standard 12-hour light:12-hour dark cycle. Rats aged 12 weeks were intraperitoneally implanted with G2 E-transmitters under isoflurane anesthesia. Rats were individually maintained in plastic home cages (276 × 445 × 204 (H) mm; CLEA Japan, Inc., Tokyo, Japan) with food and water provided ad libitum. Data were collected from 4 days after the operation. Data from 4 consecutive days were used to calculate the average activity of each individual.

### Wheel-Running Activity

Wheel-running activity was measured using the Scurry Rat Activity System (MAN80850S, MAN8611x, Lafayette Instrument, USA). Rats were housed in a standard 12-hour light:12-hour dark cycle. Male rats aged 10 to 12 weeks were individually placed in the system with free access to a home cage with food and water provided ad libitum.

### Wheel-Running Activity of Female Rats and Their Ovarian Cycles

Female rats aged 10 weeks to 12 weeks were used. Vaginal mucosal samples were obtained daily by inserting a cotton swab moistened with 0.15 M saline into the vagina. The sample was transferred to a microscope slide and stained with 0.5% toluidine blue. Phases of the ovarian cycle were identified using standard criteria (27).

### Wire Hang Test

The mesh (225 × 338 × 140 mm, with wires oriented both horizontally and vertically) was utilized for the wire hang test. Rats aged 10 weeks were analyzed in this test at ZT6. The rats were positioned at the center of the wire hang mesh and rotated slowly 180 degrees, with the duration of their ability to remain suspended on the mesh being recorded. This test was conducted twice for each individual, with an interval of 1 minute between each repetition.

### Castrated Male Rat and Testosterone Treatment

Male rats aged 12 weeks were castrated under anesthesia with isoflurane, and both testes were removed. After 3 weeks, testosterone was chronically administered to the castrated rats via 2 pieces of 20 mm long Silastic tube (internal diameter 2.0 mm; outer diameter 3.0 mm) filled with testosterone (FUJIFILM Wako Pure Chemical, Osaka, Japan) that were inserted subcutaneously in the back. The data from the second week following castration or testosterone implantation were calculated to represent the daily wheel-running activity of each individual.

### Tissues Sample Collection

Rats were sacrificed by decapitation under isoflurane anesthesia and tissues sample were rapidly collected. The gastrocnemius muscle, soleus muscle, paired testes and seminal vesicles were collected at Zeitgeber time (ZT) 6 and were measured their weight using a balance. Testes, brain, and pituitary were collected at ZT6.

### Plasma Testosterone Concentration

Rats were sacrificed by decapitation under isoflurane anesthesia and blood samples were corrected at ZT1, 6, 11.5, 18, and 24. Testosterone concentration was measured using a testosterone enzyme-linked immunosorbent assay (ELISA) kit (Cayman Chemical, 582701, RRID:AB_2895148, https://www.antibodyregistry.org/AB_2895148). Plasma samples were diluted to a 2-fold with ultrapure water. Diethyl ether (4-fold) was added and thoroughly mixed with a vortex mixer for 10 minutes and allowed to stand for 3 minutes to separate the ether and water layers. The sample was cooled with 100% ethanol cooled with dry ice. Only the lower water layer was frozen, and the upper, ether layer was collected in a new sample tube. The collected samples were incubated at 40 °C for 30 minutes to volatilize the diethyl ether; ELISA buffer was added and vortexed to dissolve the extract and use this sample for ELISA analysis. ELISA was performed according to the manufacturer's protocol.

### Hematoxylin and Eosin Staining

Testis collected at ZT6 were fixed in Bouin solution, dehydrated in an ascending ethanol series, immersed in xylene, embedded in paraffin, and sectioned at a thickness of 10 µm. The sections were dewaxed, washed with distilled water and stained with hematoxylin and eosin (3 minutes for each staining). Stained sections were washed in running tap water, dehydrated with alcohol series and with xylene, and then mounted for light microscopic observation and imaging with a digital camera.

### Luteinizing Hormone Immunohistochemistry and Morphological Analysis

Immunohistochemical detection of luteinizing hormone (LH) using rabbit antiovine LH serum (RRID:AB_3696690, https://www.antibodyregistry.org/AB_3696690, HAC-RT27-02-RBP93; a kind gift from Laboratory of Biosignal Sciences, Institute for Molecular and Cellular Regulation, Gunma University, Maebashi City, Japan) was carried out by the ImmPRESS polymerized reporter enzyme staining system (Vector Laboratories, Inc., CA, USA). Pituitary tissues fixed with 4% paraformaldehyde were dehydrated in an ascending ethanol series, immersed in xylene, embedded in paraffin (Palaplast, Sakura Finetek USA, Inc., CA, USA), and sectioned at a thickness of 10 µm. The sections of the pituitary at a site 500 µm advanced rostrally were used. The sections were dewaxed, treated with 0.5% sodium metaperiodate to block endogenous peroxidases for 15 minutes, and then incubated with TNBS (1% normal horse serum and 0.4% Triton X-100 in phosphate-buffered saline [PBS]) for 1 hour. After washing with PBS, the sections were incubated overnight in a humid chamber with the anti-LH antibody diluted at 1:50 000 in TNBS. The ImmPRESS polymerized reporter enzyme staining system was carried out with a staining kit (VECTASTAIN Elite ABC Kit Peroxidase; VECTOR Laboratories). The reactions were visualized with 0.02% 3′3-diaminobenzidine tetrachloride in 0.006% H_2_O_2_ in 50 mM Tris-HCl, pH 7.6. Stained sections were viewed under a light microscope (BX60; Olympus, Tokyo, Japan) and photographed with a digital camera (DP70; Olympus). The digital images were transformed into gray scale by Adobe Photoshop (Adobe Systems, San Jose, CA, USA), and statistical analysis was performed with cell size per pixel measured by NIH image (Scion Co., Frederick, MD, USA).

### In Situ Hybridization

Brain tissues collected at ZT6 were frozen and kept at −80 °C. ISH was performed on frozen 8-μm-thick frontal sections as described previously (23). Digoxigenin-labeled antisense and sense rat *Nmu* cRNA probes (GenBank accession no. NM_022239; positions 231-625) and rat *Nmur2* cRNA probes (GenBank accession no. NM_022275; positions 473-1270) were synthesized using a labeling kit (Roche Diagnostics GmbH, Mannheim, Germany) with SP6 or T7 RNA polymerase.

### RT-quantitative PCR

The brains collected at ZT6 were sliced with a Precision Brain Slicer (Braintree Scientific, Inc., MA, USA) and trimmed under a stereomicroscope. The area from the rostral to caudal hypothalamus was collected. Hypothalamus, pituitary and testis samples collected at ZT6 were frozen in liquid nitrogen and kept at −80 °C. RNA extraction was performed with RNAzol (Cat # R4533, Sigma-Aldrich) according to the manufacturer's protocol. Extracted RNA was treated with DNase Amplification Grade (18068015; Invitrogen) and cDNA was synthesized from the total RNA using Prime Script RT Master Mix (Takara Bio). RT-qPCR was performed using the LightCycler 96 System (Roche Diagnostics) with SYBR Premix Ex Taq (Takara Bio). The primers used for RT-qPCR are shown in Table 1. Rat Rpl19 or GAPDH expression were evaluated as an internal control. The amplicon size and specificity were confirmed by a melting curve analysis and 2% agarose gel electrophoresis.

**Table 1.** Primer sequences used for RT-qPCR

Primers	Sequences (5→3′)	GenBank accession no.
Nmu, Forward	CGT TCC TCA ACT GCA TGA GA	NM_022239
Nmu, Reverse	CCA TTG CGT GGC CTA AAT AA	
Nmur1, Forward	CAG GTG ACC AAG ATG CTA ATT GC	NM_023100
Nmur1, Reverse	ACC AGA AGC AAG GTG CAC AG	
Nmur2, Forward	CTT GAG GCG AAC AAA GTG GC	NM_022275
Nmur2, Reverse	CGA GGA CCA AGA CAA ACA GC	
Star, Forward	ACC AAG CGT AGA GGT TCC AC	NM_031558
Star, Reverse	TTC AGC TCT GAT GAC ACC GC	
Cyp11a1, Forward	TGC CTT TGA GTC CAT CAC CA	NM_017286
Cyp11a1, Reverse	AGT CTG GAG GCA TGT TGA GC	
Cyp17a1, Forward	CAG GGA GGT GCT CAT CAA GA	NM_012753
Cyp17a1, Reverse	CTG AAC ACC AAC TTC CGG TG	
Hsd3b1/2, Forward	GAC AGG AGC AGG AGG GTT TGT GG	NM_001042619
Hsd3b1/2, Reverse	CTC CTT CTA ACA TTG TCA CCT TGG CCT	
Hsd17b3, Forward	TTG GAA TGC TCC CCA ACC TG	NM_054007
Hsd17b3, Reverse	CCT CTC CGC CTT GAT TCC AT	
Hsd17b11, Forward	TGG AAC CTG AGG AGG TGG TA	NM_001004209
Hsd17b11, Reverse	TGC GCT TTA GAA CGT CCA GG	
Lhb, Forward	TCA CCT TCA CCA CCA GCA TC	NM_001033975
Lhb, Reverse	CAG AGC TAC TGA GAC GGC AG	
Fshb, Forward	CTT GGT GTG AGG GCT ACT GC	NM_001007597
Fshb, Reverse	ACA GTG GCA TTC AGT GGC TA	
Tshb, Forward	TCT GCG CTG GGT ATT GTA TG	NM_013116
Tshb, Reverse	CGG TAT TTC CAC CGT TCT GTA	
Cga, Forward	CTG TCC ATG GTC CTG CAT ATT	NM_053918.2
Cga, Reverse	CGT GAG AAA GAA GCA TCA GGA	
Prl, Forward	CAA TTC CAT GTC AGT TCT CTG CTG	NM_001433431
Prl, Reverse	CCA TCA ATG ACT GCC CCA CT	
Gh, Forward	CTC AAG AGG CTG GTG CTT TC	NM_001034848
Gh, Reverse	CAA TTC CAT GTC AGT TCT CTG CTG	
Pomc, Forward	CCA TAG ACG TGT GGA GCT GG	NM_139326
Pomc, Reverse	AGG GCT GTT CAT CTC CGT TG	
Gnrh1, Forward	GCA CTG GTC CTA TGG GTT GC	NM_012767
Gnrh1, Reverse	TCT GGG GTT CTG CCA TTT GA	
Kiss1, Forward	ACC TGT GGT GAA CCC TGA AC	NM_181692
Kiss1, Reverse	CAT GGC GAT GTT CGG CGA G	
Lhcgr, Forward	TCA CAG CTG CAG TCC CGA G	NM_012978
Lhcgr, Reverse	TGA CAG GGA GAT AGG TGA GAG AT	
Gnrhr, Forward	TGA ACG GTC TAT GAC CAG CC	NM_031038
Gnrhr, Forward	GGA AAG CTG CAG TGG GTC A	
Rpl19, Forward	ACC AAC GAA ATC GCC AAT GC	NM_031103
Rpl19, Reverse	CAA GGT GTT CTT CCG GCA TC	
Gapdh, Forward	AGG TCG GTG TGA ACG GAT TTG	NM_017008
Gapdh, Reverse	TGT AGA CCA TGT AGT TGA GGT CA	

### Isolation of Leydig Cells

The testes were sampled at ZT1 and washed twice in PBS, and then decapsulated with forceps. Decapsulated testes were incubated with collagenase solution (Collagenase, FUJIFILM Wako Pure Chemical, 034-22363/DMEM) at 34 °C for 30 minutes with shaking. After collagenase digestion, the supernatant was filtered through a 40 µm cell strainer (Corning, 352340). The filtered solutions were centrifuged at 900 rpm for 5 minutes. The pellet was resuspended in 1 mL of DMEM-BH (0.07% BSA, 25 mM HEPES/DMEM). The resuspended pellet was added dropwise onto Percoll (Cytiva, 17-0891-01) gradient solution (20%, 35% and 57% in EBSS-BH [0.07% BSA, 25 mM Hepes, pH 7.4]), and centrifuged at 2100 rpm for 20 minutes. After centrifugation, the cell layer between the 37% and 53% Percoll layers was collected as Leydig cells. After washing with PBS and centrifugation at 5000 rpm for 1 minute, the pellet was solubilized in RNAzol and stored at −80 °C for RNA extraction.

### Statistical Analysis

Data are presented as means ± SEM. Comparisons between 2 groups were performed by the Student t-test. Comparisons between 3 or more independent groups were performed by 1-way analysis of variance (ANOVA) followed by post hoc Dunnett test. Comparisons between 2 independent variables were made by 2-way ANOVA followed by the post hoc Bonferroni test. All statistical analyses were performed with GraphPad Prism 10 software (GraphPad Software, La Jolla, CA). *P* < .05 was considered statistically significant.

## Results

### Home Cage Locomotor Activity

**Figure 1.**
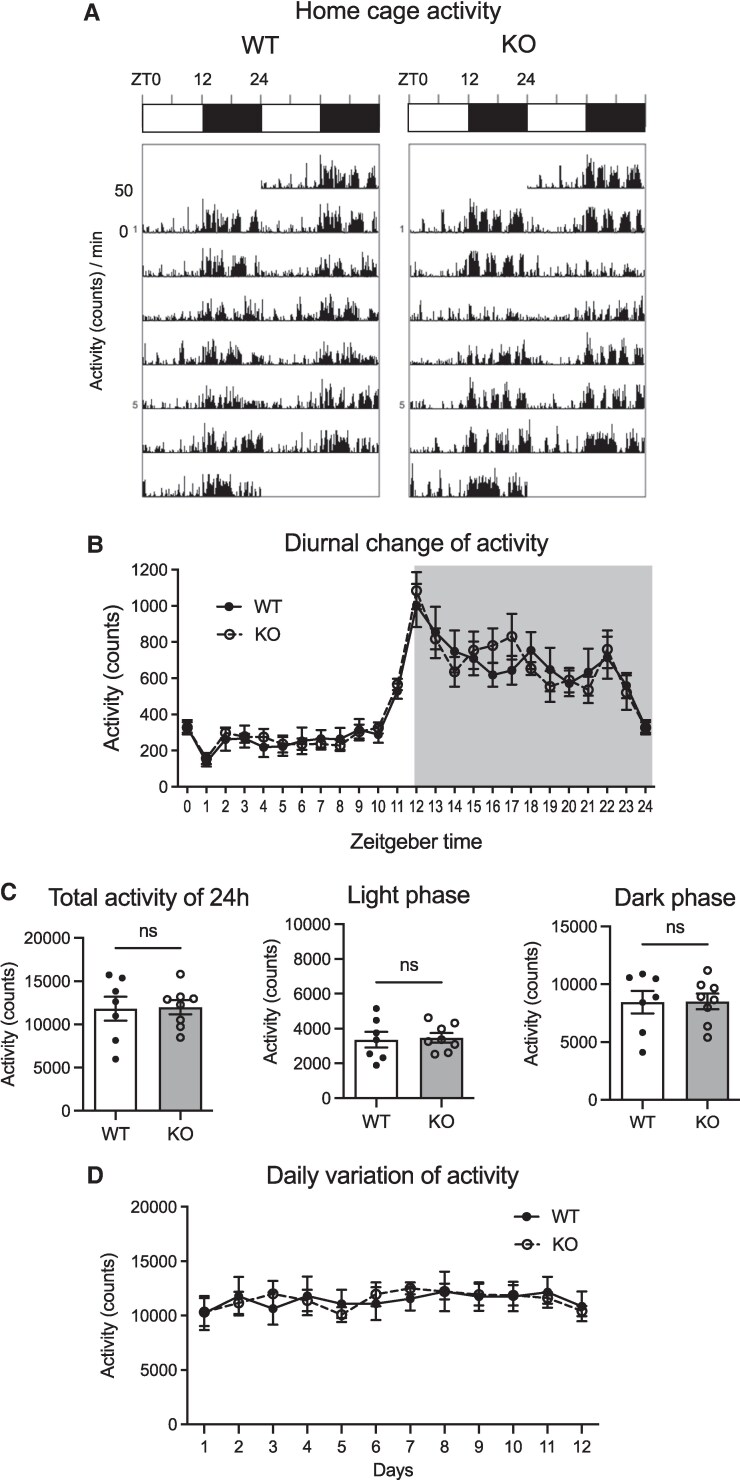
Home cage activity of male WT and NMU KO rats. Activity was measured by telemetry system in the standard rat cage. (A) Representative double-plotted histograms of the home activity patterns of WT and KO rats. (B) Hourly data of activity amount. Period of darkness is indicated by gray background. All data indicate the means ± SEM (WT n = 7, KO n = 8). (C) Activity for the full 24 hours and divided into light and dark periods. All data indicate the mean ± SEM (WT n = 7, KO n = 8). Comparisons were performed by Student's t-test (ns: not significant). (D) The daily activity amount for 12 consecutive days. All data indicate the mean ± SEM (WT n = 7, KO n = 8).

Actograms of home cage locomotor activity in 1-minute bins for 7 days showed characteristic nocturnal activity patterns of both male WT and NMU KO rats (Fig. 1A). Hourly data revealed that in both male WT and KO rats activity increased after lights were turned off and decreased prior to lights on, and there was no difference between WT and KO rats at any time point (Fig. 1B). The total 24 hours of home cage activity in NMU KO rats was equal to that in WT rats. When cage activity counts were analyzed separately for the light and dark phases, no significant difference was observed between WT rats and NMU KO rats (Fig. 1C). Figure 1D shows the variation of daily activity for 12 consecutive days; the amount of activity exhibited on a daily basis remained relatively consistent. There was no significant difference between WT rats and NMU KO rats on any day (Fig. 1D). These results indicate that NMU deficiency in male rats does not affect the amount of home cage activity.

### Effect of NMU Deficiency on Wheel-Running Activity

**Figure 2.**
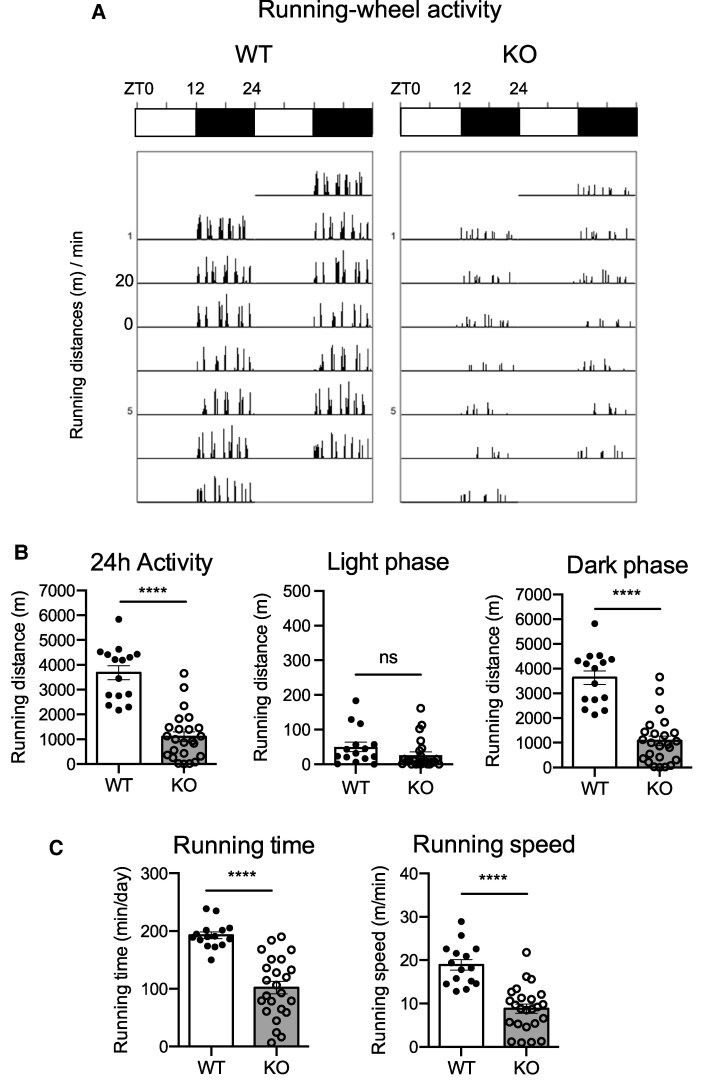

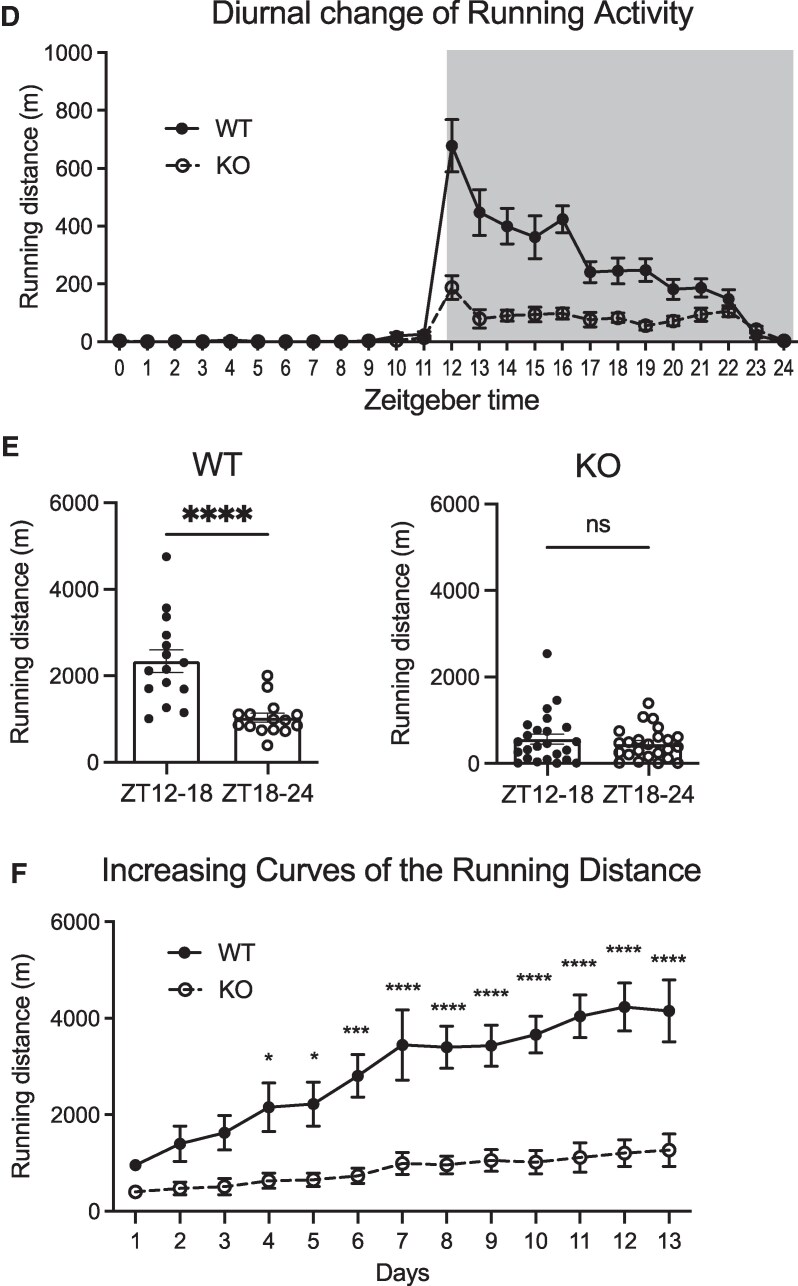
Wheel-running activity of male WT and NMU KO rats. Rats were housed in the cages with free access to a running wheel (WT n = 15, KO n = 24). (A) Representative double-plotted histograms of wheel-running activity patterns of WT and KO rats recorded in 1-minute bins for 7 days. (B) Activity for the full 24 hours and divided into light and dark periods. (C) Running time per day and average running speed. (D) Hourly running distance for 24 hours. Period of darkness is indicated by gray background. (E) Running distance summarized separately for ZT12-18 or ZT18-24. (F) The daily activity amount for 14 consecutive days from the day the rats were placed in the wheel-running system. All data indicate the mean ± SEM (WT n = 15, KO n = 24). The significance in the data for B, C, and E were determined by Student's t-test (ns: not significant, **** *P* < .0001). The significance of differences between WT and KO in the data F were determined by 2-way ANOVA and the post hoc Bonferroni test (*P < .05, ***P < .001, ****P < .0001).

Wheel-running activity is a useful measure to analyze not only typical activity but also motivated and rhythmic activity in the rodents. In this study, male rats aged 10 to 12 weeks were individually housed for 4 weeks in cages with free access to a running wheel. The data in Fig. 2A-2E were analyzed for the third week after the rats were placed in this system. Representative actograms recorded in 1-minute bins for 7 days during this week are shown in Fig. 2A. The results indicate that both WT and NMU KO rats exhibited distinct rhythmic activity, rarely showing activity during the light phase and mostly active during the dark phase, but the volume of activity was different between the groups (Fig. 2A). The daily 24-hour wheel-running distance of NMU KO rats was significantly lower than that of WT rats. There was no difference in light phase activity, but a significant difference was observed in dark phase activity (Fig. 2B). The decrease in wheel-running activity in KO rats was due to both a decrease in activity time and a decrease in running speed (Fig. 2C).

Hourly data revealed a daily change of wheel-running activity (Fig. 2D). WT rats began to run as soon as the lights were turned off and peaked at 1 hour into the dark period. After the peak, WT rats had a steady decline in wheel-running activity that gradually returned to basal levels before the lights came back on. NMU KO rats also began to run as soon as the light was turned off, but did not show a clear peak, and wheel-running activity was consistently low during the dark phase. Although WT activity was significantly higher in the first half of the dark period than in the second half of the dark period, we did not find this pattern in NMU KO activity (Fig. 2E). KO rats showed less activity than WT rats from the day they were placed in the wheel-running environment, and the difference widened with each passing day, becoming significant from the fourth day after being placed in the device (Fig. 2F).

**Figure 3.**
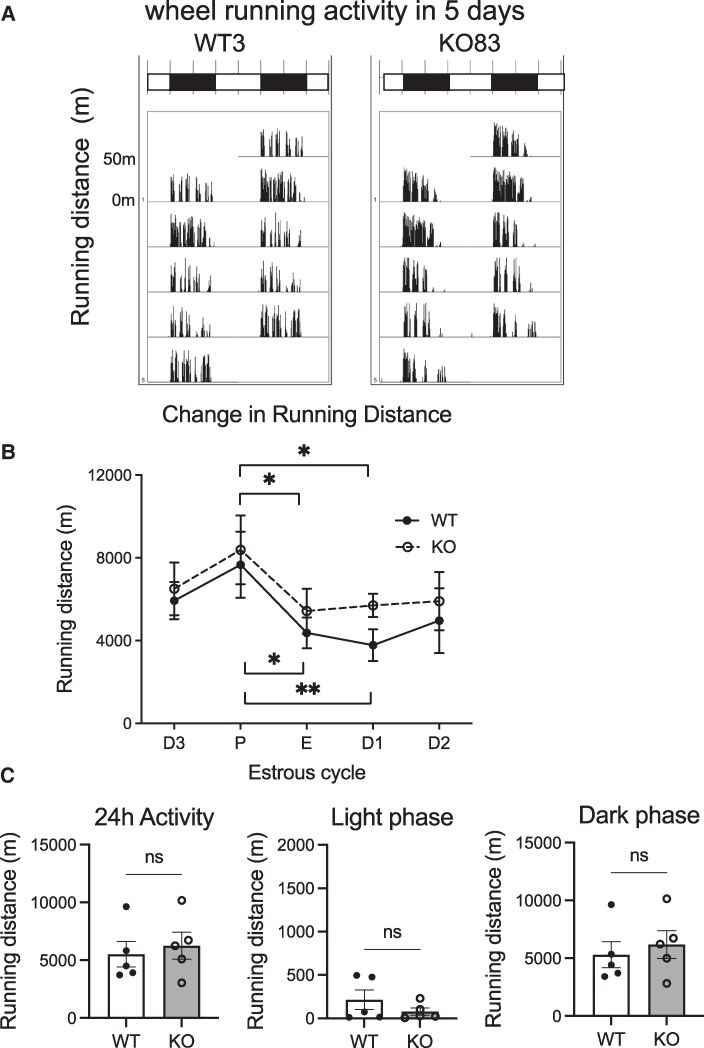
Wheel-running activity of female WT and NMU KO rats. Rats were housed in cages with free access to a running wheel. (A) Representative double-plotted histograms of wheel-running activity patterns of female WT and KO rats recorded at 1 minute each for 7 days. (B) Change in running distance between the estrous cycle phases. D, diestrus; P, pre-estrus; E, estrus. All data indicate the means ± SEM (WT n = 5, KO n = 5). Significance was determined by 2-way ANOVA post hoc test (WT_P vs WT_E *P* < .05, WT_P vs WT_D1 *P* < .01, KO_P vs KO_E *P* < .05, KO_P vs KO_D1 *P* < .05). (C) Amount of activity counts during daily 24 hours and divided into light and dark periods. Data indicate the means ± SEM (WT n = 5, KO n = 5). Significance was determined by Student's *t*-test (ns: not significant).

We also analyzed the wheel-running activity of female rats. It is well-known that estrus phase can affect activity and behavior (28, 29). In this study, female rats aged 10 to 12 weeks were individually housed for 4 weeks in cages with free access to a running wheel. All female rats displayed regular 5-day estrus cycles. Phases of the estrus cycle were identified using standard criteria (27). The average value for each individual was calculated based on the 3 estrous cycles seen between 2 and 4 weeks after the rats were placed in this system (Fig. 3B and 3C). Large differences in wheel-running activity were detected between phases of the ovarian cycle in both WT and KO female rats; it was highest at pre-estrus (Fig. 3B). In contrast to male rats, female rats demonstrated no significant difference in the wheel-running activity between WT and KO individuals in any phase of the estrus cycle (Fig. 3B), or in total daily 24-hour activity, or in light-phase and dark-phase activity (Fig. 3C).

### Wire Hang Test and Muscle Mass

**Figure 4.**
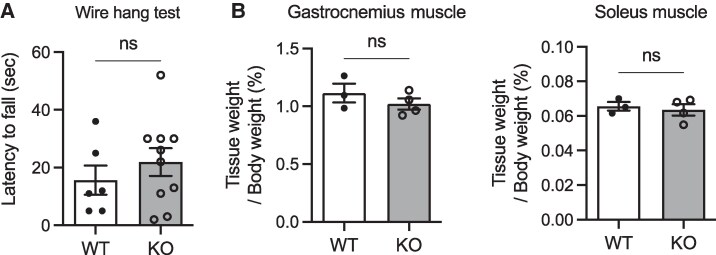
Wire hang test and muscle mass of male WT and NMU KO rats. (A) The latency to fall in the wire hang test (WT n = 6 KO n = 10). (B) Percentage of the gastrocnemius muscle and Soleus muscle content in male WT and KO rats. Data indicate the mean ± SEM (WT n = 3 KO n = 4). Significance was determined by Student's t-test (ns: not significant).

Because the reduced wheel-running activity in male NMU KO rats was potentially due to reduced motor function, a wire hang test (30) and measurement of muscle mass were performed. In a wire hang test, no significant differences were found between WT and KO rats, although there were large individual differences in time to fall (Fig. 4A). No significant difference was observed in the gastrocnemius mass and soleus muscle mass between WT and KO rats (Fig. 4B). This indicates that NMU deficiency does not affect athletic performance or muscle mass, and thus these are unlikely to be the cause of reduced wheel-running activity.

### The Effect of Testosterone Treatment on Wheel-Running Activity

Wheel-running activity in rats is reduced by castration and can be recovered with testosterone treatment in rats, suggesting that testosterone is one of the key factors in stimulating wheel-running activity (31). In this study, we also focused on reduction of testosterone as a likely cause of reduced wheel-running activity.

**Figure 5.**
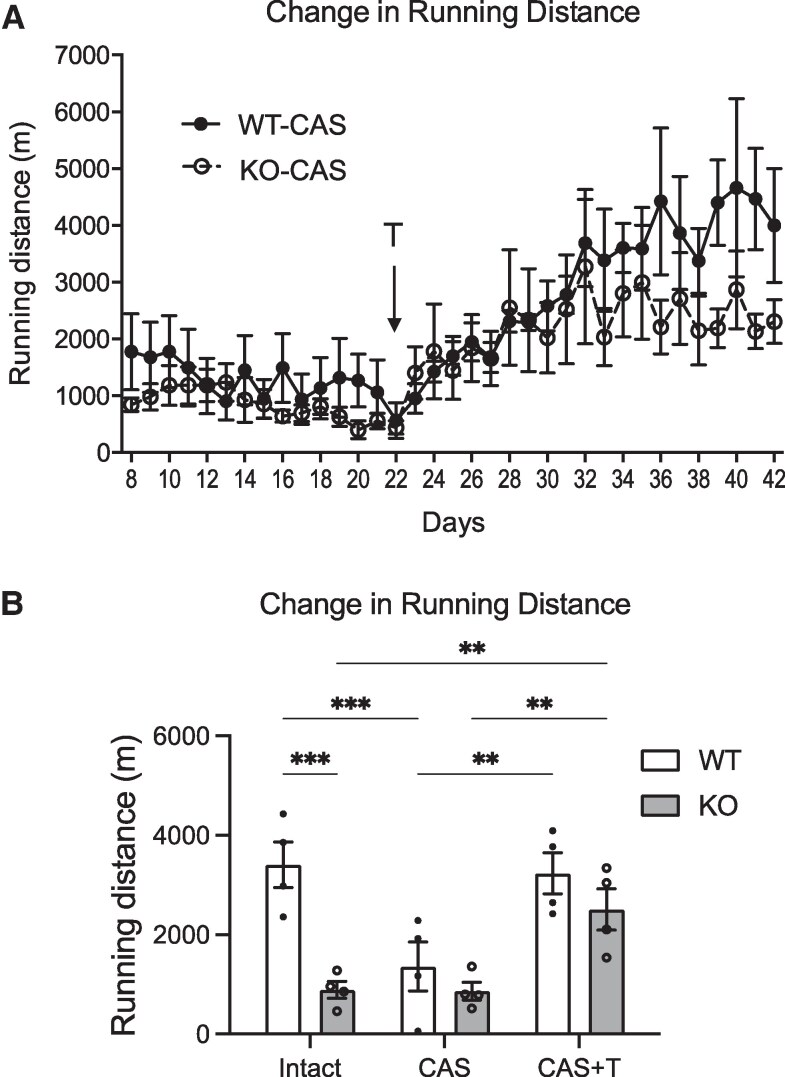
The effect of castration (CAS) and testosterone treatment (T) on wheel-running activity. (A) Daily running distance. X axis is the date since castration. Testosterone implantations (arrow, T) were treated on the day 22 in WT-CAS rats and KO-CAS rats. (B) Analysis of the running distance of the intact, CAS, and CAS + testosterone treatment (T) of the WT and KO rats. Data from the second week postcastration or testosterone implantation were calculated to represent the daily activity of each individual. Data indicate the mean ± SEM (WT n = 4. KO n = 4). Significance was determined by 2-way ANOVA and post hoc t-test (**P < .01, ***P < .001).

Both castrated male WT (WT-CAS) and KO (KO-CAS) rats exhibited low running distances (Fig. 5A). Testosterone implantation on day 22 postcastration increased the activity of both the WT and KO castrated male rats and the activity reached a plateau approximately 15 days after testosterone implantation (Fig. 5A). Plasma testosterone concentration in WT and KO rats following testosterone implants were as follows: WT_CAS + T (1.030 ± 0.103 ng/mL) and KO_CAS + T (1.061 ± 0.115 ng/mL). The initial 7-day periods following castration or testosterone treatment were designated as a recovery period and these data were not used for analysis. The data from the subsequent 7-day periods were calculated to represent the daily activity of each individual either postcastration or post-testosterone replacement. Statistical analysis revealed that the wheel-running activity in WT rats was significantly reduced by castration and then it was restored by testosterone treatment to the same level as prior to castration (no significant difference in WT_Intact vs WT_CAS + T) (Fig. 5B). On the other hand, the already low activity was not reduced further by castration in KO rats (no significant difference in KO_Intact vs KO_CAS). Testosterone treatment subsequently significantly increased activity in the NMU KO rats to the same level as in WT rats (no significant difference in WT_CAS + T vs KO_CAS + T) (Fig. 5B).

### Plasma Testosterone Level in Male WT and KO Rats

**Figure 6.**
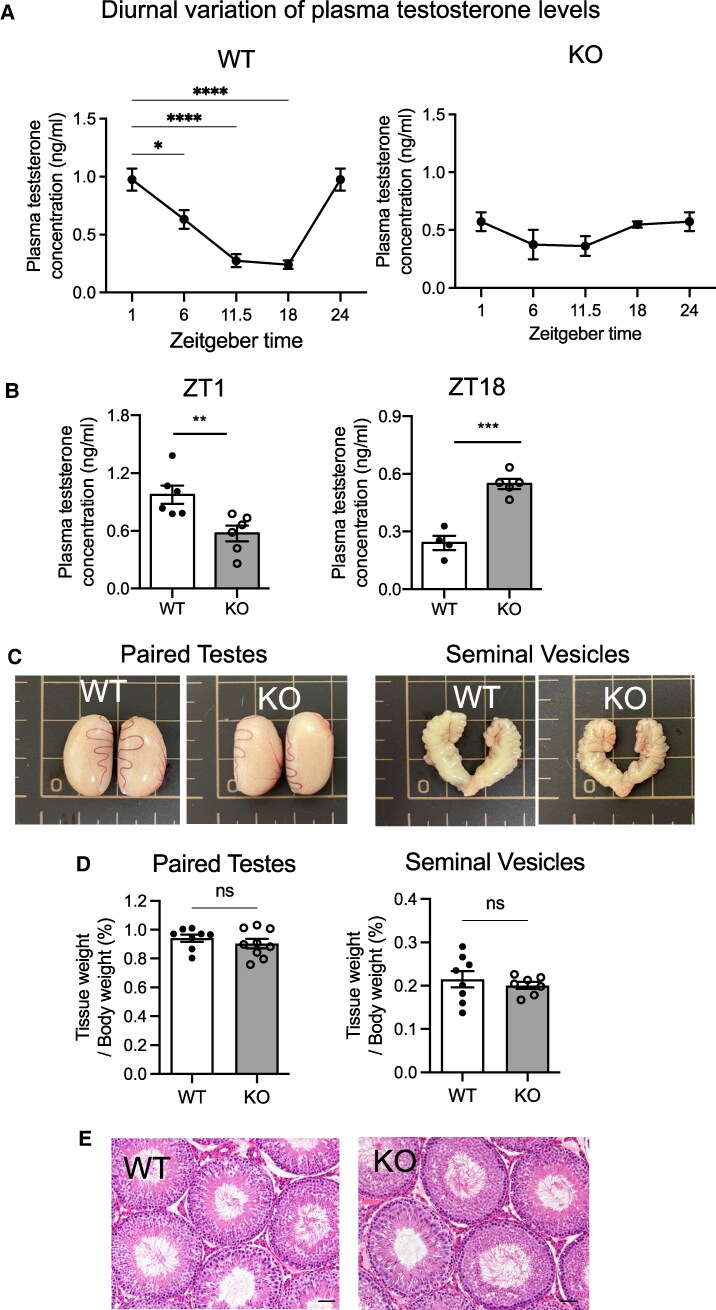
Diurnal plasma testosterone concentration of male WT and KO rats. (A) Plasma testosterone concentration at ZT1, 6, 11.5, 18 and 24. Data indicate the mean ± SEM (WT n = 6 KO n = 6). Significance was determined by 1-way ANOVA and post hoc t-test (vs ZT1, *P < .05, ****P < .0001). (B) Statistical analysis of the plasma testosterone concentration at ZT1 and ZT18 in WT vs KO. Data indicate the mean ± SEM (WT n = 6 KO n = 6). Significance was determined by Student's t-test (**P < .01, ***P < .001). (C) Representative appearance of paired testes and seminal vesicles of WT and KO rats (littermates). (D) The weight of paired testes and seminal vesicles. Data indicate the mean ± SEM (WT n = 8, KO n = 8). (E) Representative photographs of hematoxylin and eosin–stained sections of testis. Scale bar: 100 µm.

Plasma testosterone concentration was measured at ZT 1, 6, 11.5, 18, and 24. In WT rats, testosterone significantly varied over the course of a day, with a peak at ZT1 and a nadir at ZT18 (Fig. 6A). In contrast, there was no significant change in plasma testosterone levels in NMU KO rats, which remained stably low throughout the day (Fig. 6A). At ZT1, the plasma testosterone of NMU KO rats was significantly lower than that of WT rats (Fig. 6B). Conversely, at ZT18, the plasma testosterone of intact NMU KO rats was significantly higher than that of WT rats (Fig. 6B). These results indicate that the daily variation in plasma testosterone of NMU KO rats differed from that of WT rats. There was no significant difference between WT and KO rats in the mass of paired testes and seminal vesicles, and no apparent histological abnormalities the testes of the WT or KO rats (Fig. 6C-6E)

### Expression of Steroid Hormone Synthases in the Leydig Cells

**Figure 7.**
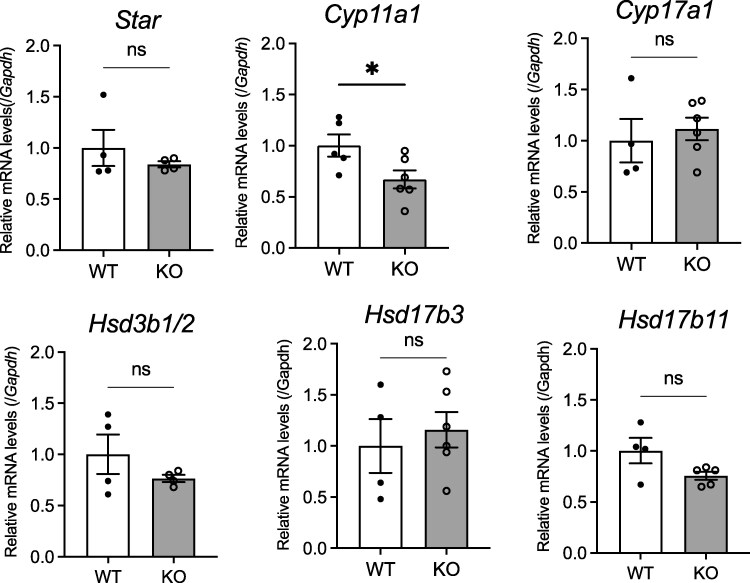
Expression levels of steroid hormone synthase in the Leydig cells. RT-qPCR analyses was performed using Leydig cells cDNA samples collected by the Percoll isolation method. All data represent means ± SEM (WT n = 4, KO n = 5). Significance was determined by Student's t-test (ns: not significant, *P < .05).

The expression of *Cyp11a1*, which is the first and rate-limiting step in the synthesis of steroid hormones in Leydig cells in testis, was significantly lower in the Leydig cells of KO rat testis (Fig. 7). There was no significant difference between WT and KO rats in the expression of *Star*, *Cyp17a1*, *Hsd3b1/2*, *Hsd17b3*, or *Hsd17b11*.

### The Effect of NMU Deficiency on the Expression of Pituitary Hormones

**Figure 8.**
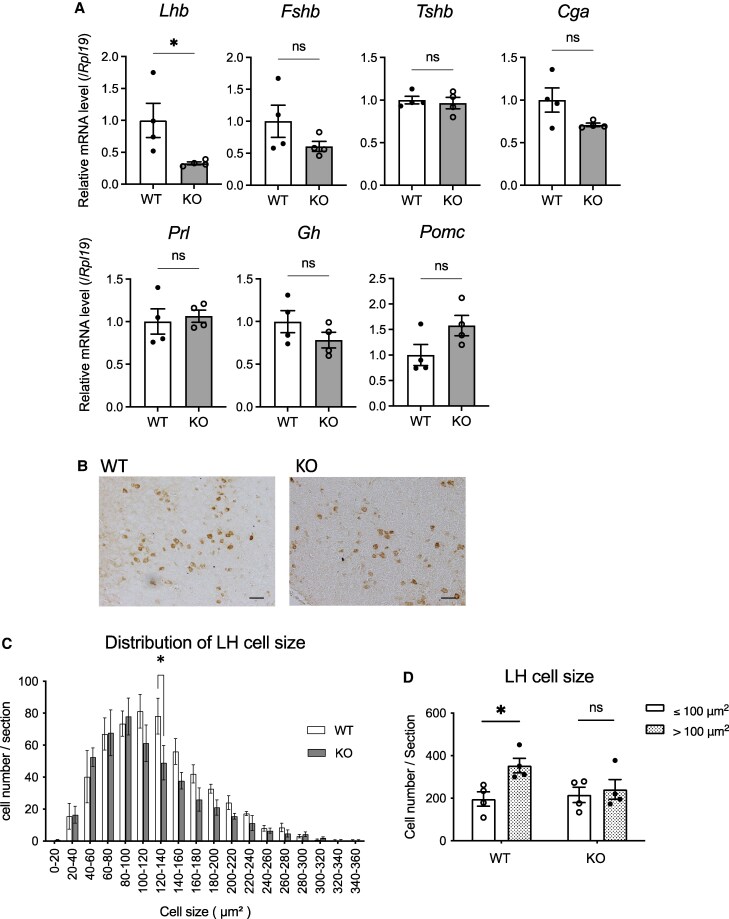
Expression levels of pituitary hormones in the pars distalis of male WT and KO rats. (A) RT-qPCR analyses was performed using the pars distalis cDNA samples. All data represent means ± SEM (WT n = 4, KO n = 4). Significance was determined by Student's t-test (ns: not significant, *P < .05). (B) The light micrographic representative image of LH-immunostaining in the pars distalis. Scale bar: 50 µm. (C) Distribution of LH cell size. All data represent means ± SEM (WT n = 4 KO n = 4). Significance between WT and KO was determined by 2-way ANOVA and post hoc t-test (*P < .05). (D) The distribution of LH cell sizes based on the analyzing cell size 100 µm^2^ as a threshold. Significance determined by Student's t-test (ns: not significant, *P < .05).

We analyzed the effect of NMU deficiency on the expression of anterior pituitary hormones in the pars distalis in male rats. The pituitary glands were collected at ZT11. Relative to WT rats the expression of *Lhb*, which encodes the LH beta subunit, was significantly lower in the KO rats (Fig. 8A). There was no significant difference between WT and KO rats in the expression of follicle-stimulating hormone beta subunit (*Fshb*), thyroid-stimulating hormone beta subunit (*Tshb*), common glycoprotein alpha subunit (*Cga*), growth hormone (*Gh*), prolactin (*Prl*), and proopiomelanocortin (*Pomc*). Furthermore, immunostaining for LH was also performed in the pituitary (Fig. 8B). The distribution of LH cell size exhibited significant difference between WT and KO rats. Notably the number of cells measuring between 120 and 140 µm^2^ was significantly lower in KO rats than in WT rats (Fig. 8C). Furthermore, upon analysis of cell size with 100 µm^2^ as a threshold, it was observed that WT rats exhibited a significantly higher prevalence of LH cells larger than 100 µm^2^ than those measuring below 100 µm^2^ (Fig. 8D). In contrast, no such discrepancy in LH cell size was detected in KO rats.

**Figure 9.**
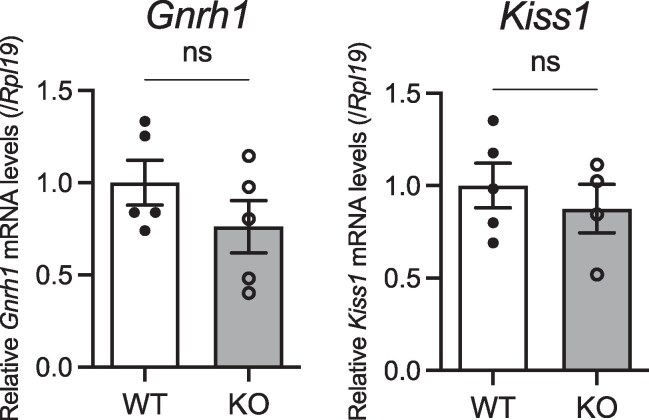
Expression levels of *Gnrh1* and *Kiss1* in the hypothalamus. RT-qPCR analyses was performed using hypothalamus cDNA samples. All data represent means ± SEM (WT n = 4, KO n = 6). Significance was determined by Student's t-test (ns: not significant).

We next investigated the potential for NMU deficiency to affect the main hypothalamic regulators for LH, GnRH, and Kiss1. RT-qPCR indicated that these did not differ between the WT and KO rats (Fig. 9).

### *Nmu*, *Nmur1*, and *Nmur2* mRNA Expression in the Hypothalamic–Pituitary–Gonadal Axis

**Figure 10.**
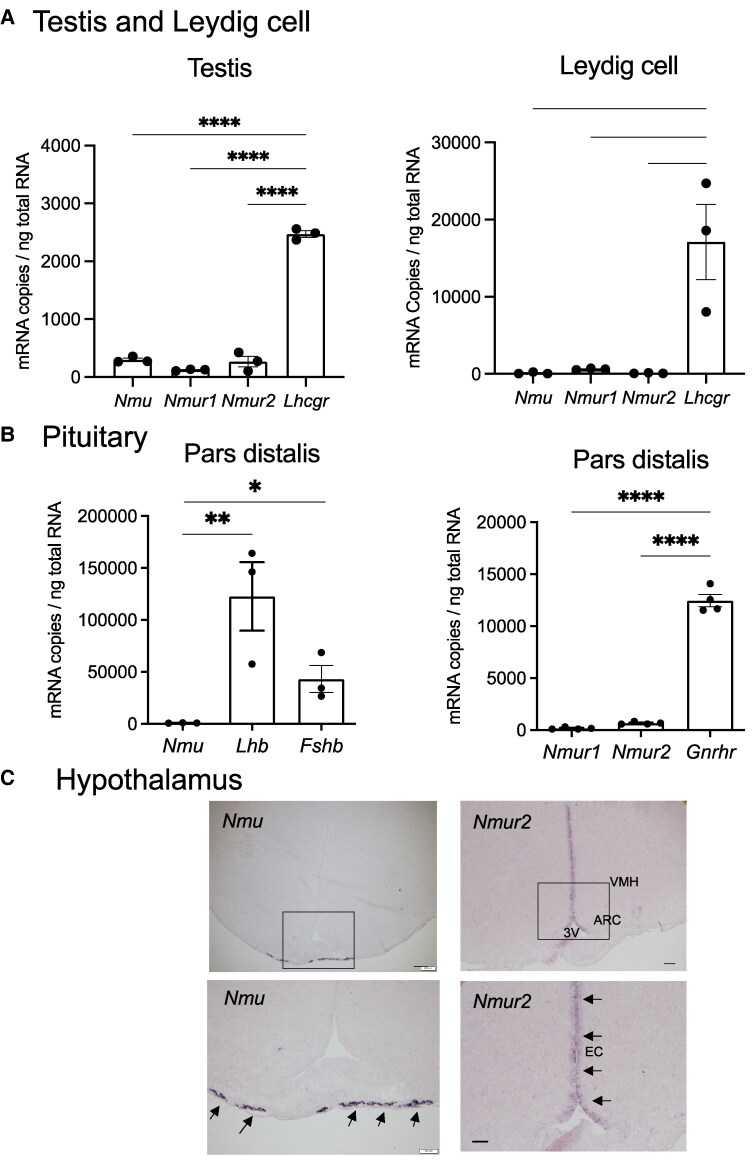
Expression of *Nmu*, *Nmur1*, and *Nmur2* in HPG axis of male WT rats. (A) RT-qPCR analysis of *Nmu*, *Nmur1*, *Nmur2*, and *Lhcgr* expression in the testis cDNA or isolated Leydig cell cDNA. All data represent means ± SEM (n = 3). Significance was determined by One-way ANOVA and post hoc t-test (**P < .01, ****P < .0001). (B) RT-qPCR analysis of *Nmu, Nmur1, Nmur2* and *Gnrhr* expression in the pars distalis. All data represent means ± SEM (n = 3). Significance was determined by 1-way ANOVA and post hoc t-test (vs *Nmu* or *Gnrhr*, *P < .05, **P < .01, ****P < .0001). (C) Localization of *Nmu* and *Nmur2* mRNA expression determined by ISH in the rat brain. Representative microphotographs of ISH staining with DIG-labeled cRNA probes in a frontal section of the WT rat brain. Positive signals are indicated by arrows in magnified images (lower). Scale bar: 100 µm. Abbreviations: VMH, ventromedial hypothalamic nucleus; ARC, arcuate nucleus; PT, pars tuberalis; 3 V, third ventricle. EC, ependymal cell layer of 3 V.

We analyzed mRNA expression of *Nmu* and its 2 cognate receptors, *Nmur1* and *Nmur2,* in the hypothalamic–pituitary–gonadal (HPG) axis of male WT rats. No positive ISH signal was obtained in the testis for *Nmu*, *Nmur1*, and *Nmur2* (data not shown). RT-qPCR analysis of whole testis revealed that the expression of *Nmu*, *Nmur1*, and *Nmur2* was detected, but their expression was significantly low compared with the LH receptor (*Lhcgr*) (Fig. 10A). In isolated Leydig cells (the primary source of testosterone) the expression of *Nmu*, *Nmur1*, and *Nmur2* was also significantly lower than that of *Lhcgr* (Fig. 10A).

No positive ISH signal for *Nmu*, *Nmur1*, or *Nmur2* was detected in the pituitary (data not shown). *Nmu* expression was however detected in the pars distalis of the anterior pituitary by RT-qPCR, but it was significantly low compared with the expression of other pituitary hormones, *Lhb* and *Fshb*. Expression of the receptors *Nmur1* and *Nmur2* was detected by RT-qPCR, but this was markedly low relative to GnRH receptor (*Gnrhr*), another functional G protein-coupled receptor (GPCR) in the pituitary.

With regard to the hypothalamus, ISH for *Nmu* detected strong expression in the pars tuberalis, with no recognizable signal in other hypothalamic regions including the preoptic area, where *Gnrh1* is expressed, and the ARC, where *Kiss1* is expressed (Fig. 10C). *Nmur2* expression was mainly observed in the ependymal layer of the third ventricle (Fig. 10C). Sense controls showed no signal (data not shown).

## Discussion

The current study demonstrated that NMU deficiency in male rats resulted in a reduction in wheel-running activity. Conversely, the amount of activity within the home cage remained unaltered, indicating that NMU does not simply regulate overall activity levels but is involved in the control of some specific activities—in this case, the motivated behavior of wheel running. We did not observe a reduction in wheel-running activity in female NMU-deficient rats, but this activity varied according to estrus stage similarly in WT and KO female rats.

Previously, NMU has been demonstrated to increase home cage activity in rats. Intracerebroventricular administration of NMU immediately increased gross locomotor activity in home cages as measured by light-beam detectors (25). In another report, chronic NMU injection directly into the PVN and ARC, where NMUR2 expression is reported (22), significantly and dose dependently increased physical activity in home cages (26).

In the present study a reduction in the amount of wheel-running activity in males was observed resulting from NMU deficiency, but there was no alteration in home cage activity. To our knowledge, this study is the first to report involvement of NMU in a specific type of motivated activity. This finding raises the question of what distinguishes wheel-running activity from that of ordinary physical activity. Both running wheel and home cage activity sensors are often used to evaluate “activity” as a whole. However, they are distinct evaluation methods and measure distinctly different activities (32). Wheel running indicates “stereotyped running activity,” while movement in the home cage consists of the general physical activity in life, including walking, running, climbing, rearing, and maintenance activities such as grooming and eating (32, 33).

Wheel-running activity in rodents has been a long-standing component of activity research due to the rodents' apparent affinity for this behavior (33). However, there is a lack of consensus regarding the precise definition of the type of behavior that “wheel-running activity” is. When running wheels are placed in nature for wild animals to access freely, they have been observed to be used spontaneously by rodents such as mice, shrews, and rats, even though no extrinsic reward is provided. This suggests that wheel-running activity is an elective behavior that is actively sought by rodents and seems to be self-motivated and intrinsically rewarding (34). Moreover, a variety of exogenous and endogenous factors has been reported to influence wheel-running activity. These include, food deprivation (35, 36), temperature, photoperiod, stimulant drug, age, sex, stress, neuro/endocrine status, and addiction (33). Overall, wheel-running behavior appears to be motivated in a fashion that is distinct from general daily maintenance behaviors.

Overall female rats tend to exhibit more wheel-running activity than male rats. In female rats, the pattern of wheel-running activity varies throughout their estrous cycle, with the greatest activity occurring during estrus (in previous studies) and proestrus in our study (Fig. 3) (29, 37). In male rats, castration can dramatically decrease wheel-running activity, and this effect can be reversed by subsequent testosterone treatment (38). Additionally, estradiol treatment of the castrated male rats caused a greater increase in wheel-running activity (39). It was also reported that wheel-running activity of both male and female rats was decreased permanently to a very low level by removal of the gonads, and this effect was reversed via testicular grafts (38). These studies demonstrate the significance of sex steroids in the induction of wheel-running activity.

In the present study, we demonstrated that plasma testosterone in the male WT rats showed significant diurnal change. In contrast, the male KO rats showed no such variation and had generally low testosterone throughout the day. Taken together with previous studies on the significance of sex steroids on wheel-running activity, our findings suggest that the low wheel-running activity in the male KO rats was caused by abnormal testosterone production. Further, we demonstrated that castration markedly diminished wheel-running activity in male WT rats to a degree similar to that observed in intact male KO rats, and this reduction was reversed by testosterone treatment. These results support the importance of testosterone as a modulator of wheel-running activity. We also hypothesize that the enhancement of wheel-running activity is influenced by testosterone concentrations exceeding a certain threshold value of somewhere between 0.5 and 1.0 ng/mL, which is seen diurnally in WT rats and not in KO rats, but is exceeded in KO rats implanted with testosterone. It is also noteworthy that male KO rats exhibited no additional decline in the wheel-running activity following castration. It is likely that this is because the wheel-running activity of intact KO rats was already low because of an abnormal pattern of testosterone production. Moreover, the activity of the KO rats was reinstated by testosterone administration to a level similar to that observed in intact WT rats and castrated WT rats treated with testosterone. This indicates that the abnormality of testosterone production observed in male NMU KO rats is at least part of the underlying cause of the observed reduction in wheel-running activity.

Daily fluctuations in testosterone are believed to play an important role in maintaining healthy and motivational behavior (40, 41). It is well documented that a loss of the daily peak in testosterone due to disruption of the overall circadian rhythm can result in a reduction in motivation and a decline in activity levels in humans. Taken together, our data suggest that male KO rats exhibit a reduction in their motivation to run in wheels as a result of the loss of a daily peak in testosterone.

In this study, it is interesting to note that in the pituitary of male KO rats, there was a significant decrease in *Lhb* mRNA expression and a similar, though less pronounced, trend in FSH expression. Moreover, a diminution in the size of LH-producing cells was also observed. LH cells exhibited a different size distribution between WT rats and KO rats, with the KO rats showing a higher proportion of relatively smaller LH cells than the WT rats. These findings indicate that LH production was impaired in male KO rats, which likely explains the observed abnormalities in testosterone production.

Thus, our data raise the question of why NMU KO caused the reduction in testosterone. To investigate the mechanism of action of NMU, it is essential to analyze the expression sites of NMU and its receptors. In rats, NMU and its receptors have been reported to be expressed in various tissues of the brain and peripheral organs determined by PCR, qPCR, or ISH, but the results lack consistency across reports. In the present study on rats, we analyzed the expression levels of *Nmu*, *Nmur1*, and *Nmur2* in the HPG axis. Our data indicate that the HPG axis in NMU KO rats is functional but that, unlike WT rats, KO rats do not produce a daily peak in testosterone that is coincident with increased running activity. We interpret this finding as a result of NMU being an important regulator of motivated activity, but alternatively this could be an indirect effect of NMU KO via GnRH, LH, or gonadal steroidogenic enzymes. In the testes and in isolated Leydig cells, *Nmu*, *Nmur1*, and *Nmur2* were detectable by RT-qPCR. However, when compared with the expression of the LH receptor, which is a GPCR known to be expressed in Leydig cells, their expression appeared insufficient to exert functional activity. Therefore, it is unlikely that NMU acts directly on or within the testes, and it is therefore unlikely that NMU KO had a direct effect on testicular testosterone production.

In the pars distalis of the pituitary, even though the expression of *Nmu*, *Nmur1*, and *Nmur2* was detected by RT-qPCR, it remains unclear whether NMU acts directly on LH-producing cells to cause these effects. In particular, we observed NMU expression to be thousands of times lower than that of other pituitary hormones, such as *Lhb* and *Fshb*. These data raise doubts about a functional role for NMU as a pituitary hormone, even though its expression was detected. Similarly, although *Nmur1* and *Nmur2* expression was detected in the pars distalis, their levels were significantly lower than that of *Gnrhr*, which is known to be expressed in LH-producing cells. On the other hand, low expression of these ligands and receptors does not necessarily mean that they are not functional. Indeed, NMU has been reported to be expressed in some cell populations of rat pituitary corticotropes in a previous study (42, 43) and paracrine signaling from such rare populations could still exert significant effects on neighboring cell types. Based on these results, it is difficult to conclude that the abnormalities in the pituitary LH cells of male KO rats were a direct effect of NMU.

In contrast to the pars distalis and in agreement with earlier studies, NMU exhibited high levels of expression in the pars tuberalis in the rat, and *Nmur2* demonstrated high levels of expression in the epithelial cell layer of the third ventricle. These observations were evident through ISH methods, which have lower sensitivity than RT-qPCR. Given the anatomical location of NMU and its receptor, it is highly possible that the NMU produced in the pars tuberalis acts on NMUR2 in tanycytes, which are located in the epithelial cell layer of the third ventricle. Tanycytes are glial cells characterized by distinctive morphologies, extending their cellular projections to the surrounding neuronal nuclei, DMH, VMH, and ARC, as well as to the median eminence (44, 45). Some tanycyte projections reach the outer layers of the median eminence, where they physically interfere with GnRH nerve terminals and have been reported to regulate the level of GnRH secretion into the pituitary portal vein at axon terminals (46, 47 ). Tanycytes also extend projections to the VMH, suggesting a potential role in regulating VMH functions (45). Notably, Narita et al reported that the VMH plays a crucial role in estrogen-induced increases in locomotor activity (48). Further research is necessary to determine whether these pathways are affected in NMU KO rats and to explore potential underlying mechanisms.

This study reveals a novel phenotype in NMU-deficient rats. They exhibit a lack of peak and nadir circulating testosterone over the daily cycle and a reduction in wheel-running activity. At this point, it is unclear whether NMU in rats influences only wheel-running activity, or other hedonistic behaviors, such as food intake preferences, social interactions, grooming behaviors, and sexual behaviors. Our data indicate that NMU in rats is likely involved in the regulation of at least 1 motivated behavior, and not general maintenance behavior.

## Data Availability

All data generated and analyzed during this study are included in this published article.

## Abbreviations

ANOVA: analysis of variance
ARC: arcuate nucleus
DMH: dorsomedial hypothalamic nucleus
ELISA: enzyme-linked immunosorbent assay
FSH: follicle-stimulating hormone
HPG: hypothalamic–pituitary–gonadal
KO: knockout
ISH: in situ hybridization
LH: luteinizing hormone
NMU: neuromedin U
PBS: phosphate-buffered saline
qPCR: quantitative polymerase chain reaction
PVN: paraventricular nucleus
VMH: ventromedial hypothalamic nucleus
WT: wildtype; ZT, Zeitgeber time
